# Curcumin alleviates broiler liver damage caused by polybrominated diphenyl ether concurrent with restoring gut microbiota, reducing liver inflammation and endoplasmic reticulum stress

**DOI:** 10.5713/ab.250966

**Published:** 2026-06-24

**Authors:** Xiyue Zhang, Fan Zhai, Xueqin Li, Xin He, Congcong Jiang, Wanyu Shi, Shixia Zhang, Jilang Tang

**Affiliations:** 1 College of Veterinary Medicine, Hebei Agricultural University, Baoding, China

**Keywords:** BDE-209, Broiler, Curcumin, Endoplasmic Reticulum Stress, Inflammation, Microbiota

## Abstract

**Objective:**

The purpose of the present study was to evaluate the protective effects of curcumin (Cur) against broiler liver damage caused by exposure to polybrominated diphenyl ether (BDE-209).

**Methods:**

Four groups of 96 one-day-old broiler chicks were randomly assigned to the control group, the BDE-209 (0.4 g/kg) group, the BDE-209 (0.4 g/kg)+Cur (0.3 g/kg) group, or the Cur (0.3 g/kg) group. Following a 42-day treatment period, intestinal microbiota, hepatic inflammatory response, and endoplasmic reticulum stress were evaluated.

**Results:**

BDE-209 caused severe intestinal damage, including shortened villi, increased crypt depth, disruption of tight junction structures, and ultrastructural abnormalities. Cur treatment alleviated these pathological changes, restoring tight junction protein expression and improving intestinal barrier function. Cur also cured BDE-209-induced gut microbiota dysbiosis by increasing beneficial bacteria such as Lactobacillus and reducing harmful bacteria including Proteobacteria. Moreover, Cur lowered intestinal levels of key pro-inflammatory cytokines. BDE-209 caused significant liver damage, including histological lesions, hepatocyte ultrastructural damage, elevated blood liver enzymes, and oxidative stress. Cur improved liver function, lowered NF-κB and MAPK signaling pathways, and reduced the expression of endoplasmic reticulum stress-related proteins and genes.

**Conclusion:**

Cur alleviated BDE-209-induced liver damage in broilers while also restoring gut microbiota and reduction of inflammation, and endoplasmic reticula stress.

## INTRODUCTION

In the building, plastics, textile, and electronics sectors, polybrominated diphenyl ethers (PBDEs) are a common class of persistent organic pollutants (POPs) that are used as flame retardants [1]. These PBDEs are readily volatilized and seep out of the plastics to contaminate the surrounding area [2]. Studies have shown that BDE-209 is found in soil, air, indoor dust, and food, and can accumulate via the food chain in marine and terrestrial animals [3], leading to endangering animal, human, and ecological health [4]. Studies have shown BDE-209 can be passed to chicken eggs and accumulated in the liver of hens [5]. The concentrations of BDE-209 in chicken liver ranged from 11.56 to 443.8 μg/kg [6]. Notably, BDE-209 not only accumulates in the liver but can also cause damage to the liver, such as oxidative stress and infiltration of inflammatory cells [7]. Despite the severity of BDE-209-induced liver damage, little research has been done on its effects on broilers. Therefore, to develop effective strategies for protecting broiler liver health after BDE-209 exposure, it is imperative to investigate the mechanisms responsible for BDE-209-induced liver damage in broilers.

Hepatocytes have active metabolic functions and many endoplasmic reticula (ER), where the ER is involved in many cellular processes. Endoplasmic reticulum stress (ERS) is a common consequence of protein metabolism issues and exposes liver cells to many external stimuli, leading to the accumulation of reactive oxygen species (ROS), the activation of NF-κB and MAPK pathways, and the inflammatory response [8]. In addition, intestinal inflammation genes are frequently up-regulated in conjunction with the increased expression of ERS-related factors [9]. Researches have shown ERS is crucial in liver inflammatory conditions and acute injury [10]. Therefore, we posit a correlation between inflammation and BDE209-induced liver damage.

Curcumin has numerous biological properties including antioxidant, anti-inflammatory, immune-modulatory, anti-microbial, and nephroprotective [11]. It has been shown to increase beneficial intestinal bacteria and limit the growth of opportunistic pathogens, thereby delaying the onset and progression of diseases [12]. The protective effect of curcumin on the liver has been extensively studied. It can improve the structure of the liver and reduce pathological damage. Despite its protective and restorative effects, it is unknown if curcumin protects against liver damage induced by BDE-209. Given that the liver’s portal system connects the gut and liver, and the tight junction (TJ) proteins control intestinal permeability. This prevents pathogenic intestinal bacteria from entering the bloodstream and the liver. Hepatic steatosis and fibrosis can be driven by gut dysbiosis, which increases intestinal permeability and subsequent hepatic exposure to toxins [13]. However, the effects of gut microbiota and the intestinal barrier on BDE-209-induced liver damage remain unclear, and curcumin’s impact on liver damage through the gut needs further investigation. To this end, the effects of curcumin on the intestinal microbiota structure, intestinal barrier function, liver ERS, and inflammation in broilers exposed to BDE-209 were measured.

## MATERIALS AND METHODS

### Animals and experimental design

96 healthy one-day-old broilers were obtained from Dazhuang Qingmu Farm (Baoding, China) and housed in a clean, well-ventilated chicken house under hygienic conditions. Starting at one week of age, the temperature was maintained at 35°C and then reduced by 3°C every week until reaching 25°C, with relative humidity kept at 40%–50%. Sterilized feed and clean water were provided ad libitum. The broilers were randomly divided into four groups. These were defined as the Control group (fed with basic feed daily); the BDE-209 group (daily addition of 0.4 g/kg BDE-209 to the basic feed); the Cur group (daily addition of 0.3 g/kg curcumin to the basic feed); and the BDE-209+Cur group (daily addition of 0.3 g/kg curcumin and 0.4 g/kg BDE-209 to the basic feed). The dose of BDE-209 (0.4 g/kg) was selected based on a previous study [7], that induced sub chronic liver injury in broilers. The dose of curcumin (0.3 g/kg) was selected based on our preliminary dose-finding experiment (0.1, 0.2, 0.3, 0.4 g/kg), which showed that 300 mg/kg produced optimal hepatoprotection against BDE-209-induced liver injury without adverse effects, consistent with previously reported safe and effective dosages in broilers [14]. The experiment lasted 42 days. Body weight gain, feed intake, and feed conversion ratio were recorded at 1, 21, and 42 days. At the end of the trial, blood was aseptically collected from the wing vein and centrifuged (1,006×g, 5 min) to obtain serum. Broilers were then euthanized by cervical dislocation. Liver and intestinal tissues were collected for subsequent analysis.

### Histopathological analysis

Liver and intestinal tissues were fixed in 10% formalin (>24 h), dehydrated through a graded ethanol series and xylene, and embedded in paraffin. Sections were cut using a Leica rotary microtome (Leica), stained with hematoxylin-eosin (H&E), and scanned with a Leica Aperio CS2 slide scanner (Leica). A blinded pathologist evaluated liver injury according to Sneed et al. [15], scoring necrosis, hemorrhage, sinusoidal inflammatory infiltrate, and parenchymal inflammatory infiltrate (0–4 each; total score summed). Intestinal injury was scored using the Shen et al. [16] method.

### Transmission electron microscope observation

Tissues were fixed in 2.5% glutaraldehyde solution for 2–4 hours and then fixed for another 2 hours in 0.1 M PBS containing 1% osmotic acid. Following a succession of dehydration procedures, the specimens were implanted in epoxy resin. The intestine and liver sections were cut into 50–60 nm serial sections, stained with lead citrate and 2% uranyl acetate, and examined with transmission electron microscope (HITACHI).

### Determination of liver function enzyme activity and inflammation factors

Using commercial kits (Nanjing Jiancheng Technology), the activities of liver function enzymes including aspartate aminotransferase (AST), alanine aminotransferase (ALT), lactate dehydrogenase (LDH), alkaline phosphatase (ALP) and gamma-glutamyl transferase (GGT) were determined in the serum. Using enzyme-linked immunosorbent assay (ELISA) kits (Jiangsu Jingmei Biotechnology) and a microplate reader (Bio-Rad Life Medical Product), the levels of TNF-α, IL-1β, and IL-6 were quantified in the intestinal tissues. The manufacturer’s guidelines were followed.

### 16SrRNA sequencing of fecal microbiota

Total genomic DNA was extracted from stool samples using the OMEGA Soil DNA Kit. DNA quality and purity were assessed via agarose gel electrophoresis and a NanoDrop NC2000 (Thermo Fisher Scientific) spectrophotometer. The V3-V4 region of the bacterial 16S rRNA gene was amplified by polymerase chain reaction (PCR) using primers 338F (5′-ACTCCTACGGGAGGCAGCA-3′) and 806R (5′-GGAC TACHVGGGTWTCTAAT-3′). Alpha diversity was evaluated in QIIME. Beta diversity analysis was performed based on UniFrac distances and visualized via principal coordinate analysis (PCoA). Microbial community composition was analyzed and visualized at the phylum and genus levels. Inter-group differences were assessed using PERMANOVA and LEfSe.

### Detection of antioxidant markers in the liver

Liver tissue (0.1 g) was homogenized in 0.9 mL of PBS to prepare a 10% tissue homogenate, then centrifuged (12,000×g, 15 min) The supernatant was collected for assays of viability of glutathione (GSH), malondialdehyde (MDA), catalase (CAT), and total antioxidant capacity (T-AOC) with commercial kits (Nanjing Jiancheng Technology).

### Reverse transcription real-time quantitative polymerase chain reaction assay

Total RNA was isolated with the TransZol Up Plus RNA kit (Beijing All-Style Gold Biotechnology). The A260/A280 ratio was analyzed using the MicroDrop spectrophotometer (BIO-DL). The ReverTra AceTMqPCR RT Master Mix was used to synthesize complementary DNA, following the manufacturer’s protocol. The QuantStudioTM7 Flex Real-Time PCR machine and TB Green Premix Ex Taq II from Applied Biosystems were used. Gene expression was normalized to GAPDH and calculated using the 2^−ΔΔCt^ method. Primers synthesized by Sangon Biotechnology are listed in Table 1.

### Western blotting analysis

Samples were treated by radioimmunoprecipitation assay buffer (Solarbio), and a BCA protein assay kit (Solarbio) was used to measure the protein content. The protein samples were transferred to nitrocellulose membranes after being subjected to SDS-PAGE gel electrophoresis, accompanied by blocking the membranes with skim milk powder in PBST. The primary antibody (Table 2) was left to incubate at 4°C overnight. Thereafter, the membrane was cleaned with PBST and then incubated for one hour with a secondary antibody coupled with horseradish peroxidase. Enhanced chemiluminescence reagents (Suzhou Xinseimei Biotechnology) were used to identify protein bands, which were then analyzed using NIH Image J software (ver. 1.53). Diluted concentrations of antibodies are shown in Table 2.

### Immunohistochemical staining

Liver sections were dewaxed, rehydrated, and treated with 10% H_2_O_2_ to quench endogenous peroxidase. After PBS washing and blocking with normal goat serum, the sections were incubated overnight at 4°C with primary antibodies against GRP78, TRAF2, or CHOP. Following PBS rinsing, an HRP-conjugated secondary antibody was applied. The sections were then washed, developed with DAB, and examined under a fluor microscopy (Zeiss). Images were acquired and quantitatively analyzed using ImageJ (ver. 6.0).

### Statistical analysis

The gathered data were reported as mean±standard deviation and subjected to a one-way ANOVA in SPSS (ver. 25.0). If the p-value fell below the 0.05 threshold, differences were considered statistically significant. All graphs were created in GraphPad Prism (ver. 8.0).

## RESULTS

### Effect of curcumin on the performance of broilers exposed to BDE-209

Table 3 shows the growth of the broilers. Significant reductions in daily feed intake were observed among broilers in the BDE-209 group (p<0.05) compared to the control group. Compared to the control group, the BDE-209 group showed significantly lower (p<0.01) average daily weight gain and body weight by day 42. The Cur broiler group showed an increase of 9.06 g in average daily gain and 354.2 g in body weight compared to the BDE-209 group (p<0.01). In the BDE-209+Cur group, the broiler’s average daily growth and body weight increased by 6.24 g and 244.1 g, respectively (p<0.05). The broiler liver weight and liver index in the BDE-209 group increased by 6.1 g and 0.58%, respectively, compared to the control group (p<0.01) and increased by 2.4 g and 0.2%, respectively in the BDE-209+Cur group (p<0.05). The broilers in the BDE-209+Cur group showed a decrease of 0.38% (p<0.05) in the liver index and 3.7 g (p<0.01) in liver weight compared to the BDE-209 group. Liver weight and index were substantially lower in Cur group broilers than in the BDE-209 group.

### Effect of curcumin on intestinal pathological changes induced by BDE-209

In this study, we investigated the effect of curcumin on a BDE-209 exposure-induced liver and intestinal injury model in broilers (Figure 1A). H&E staining results showed that BDE-209 damaged the intestinal tissue of broilers significantly after 42 d exposure (Figures 1B–1E). Compared to the control group, significant damage was observed in the BDE-209 group along with shorter intestinal villi length, significantly increased crypt depth, significantly lower villus height/crypt depth (p<0.01), and thinner intestinal wall with a loose structure. These phenomena were alleviated in curcumin-treated broilers, where the BDE-209+Cur group showed mild villus damage, improved villus height, crypt depth, and height/crypt depth ratio (p<0.01), and increased intestinal wall thickness. In the Cur group, the mucosal layer of intestinal tissue was intact, enterocytes or intestinal glands were abundant and closely arranged, and the length of the intestinal villi was longer. Subsequently, samples were scored according to the observed intestinal pathological damage (Figure 1F) (there was no damage score in the control and Cur groups). The intestinal scoring of the BDE-209 group varied between 4 and 5, which was significantly higher than that of the control group (p<0.01), and while the intestinal score of the BDE-209+Cur group was much lower compared to the BDE-209 group (p<0.01), it was still higher than that of the control group (p<0.05). The intestinal epithelium morphology of the control group exhibited consistently aligned microvilli, entire TJ structures, and undamaged organelles (Figure 1B). The BDE-209 group had a lower microvilli count, lodging and shedding, and uneven microvilli length and arrangement along with irregular TJ structures, enlarged mitochondria, and the appearance of vacuoles in between epithelial cells. In the BDE-209+Cur group, the microvilli of the intestinal epithelium were regular and the TJ structure tended to be intact. Moreover, vacuoles decreased or disappeared in comparison with the broilers in the BDE-209 group.

### Effect of curcumin on intestinal barrier dysfunction in BDE-209-induced broilers

When compared to control broilers, BDE-209 treatment significantly decreased the intestinal expression levels of the barrier-forming TJ proteins ZO-1, Occludin, and Claudin-1 (Figures 1G–1J, p<0.01). The intestinal barrier disruption in broilers treated with BDE-209 was supported by the results of mRNA expression analysis of ZO-2, Claudin-3, and Occludin (Figures 1K–1M). Following curcumin treatment, the TJ protein expression level in the BDE-209+Cur group increased significantly (p<0.05) compared to the BDE-209 group, and the mRNA expression levels of Occludin and *Claudin*-3 (p<0.05) and *ZO*-2 (p<0.01) increased correspondingly.

### Effect of curcumin on the changes in intestinal inflammatory factors induced by BDE-209

As demonstrated in Figures 1N–1P, the BDE-209 group had considerably higher amounts of IL-6, IL-1β, and TNF-α compared to the control group (p<0.01). After curcumin treatment, the BDE-209+Cur group’s TNF-α, IL-1β, and IL-6 concentrations were much lower than those of the BDE-209 group (p<0.01). Furthermore, the quantities of these three inflammatory factors were markedly decreased by curcumin addition alone (p<0.01).

### Effect of curcumin on intestinal microflora disorders in BDE-209-induced broilers

We found that, whereas BDE-209 treatment significantly disrupted the gut microbiota, curcumin treatment partially restored the stability of the gut microbiota. The alpha diversity of the three groups did not differ significantly (Figure 2A), but Curcumin affected the gut microbiota’s beta diversity, according to PCA (Figure 2B). The BDE-209 group and the control group were more clearly defined. The samples from the BDE-209+Cur group were more similar to the control group than the BDE-209 group, suggesting that the addition of curcumin to the feed improved the beta diversity of the gut microbiota. In contrast to the control group and the BDE-209+Cur group, which displayed a lower relative abundance of *Proteobacteria* and a greater relative abundance of *Firmicutes*, the BDE-209 group had a higher relative abundance of *Proteobacteria* (Figure 2C). The BDE-209 group also showed a decrease in the relative abundance of *Lactobacillus*, *Oscillospira*, and *Ruminococcus* (Figure 2D). This group also had an increased relative abundance in *Bilophila* and *Subdoligranulim*, while the control group and the BDE-209+Cur group showed the opposite. Lefse and LDA data (Figures 2E, 2F) demonstrated that the gut microbiota of the BDE-209 group differed significantly from that of the control group, with a decrease in *Lactobacillus* and an increase in pathogenic bacteria, including *Proteobacteria* and *Bilophila*. The BDE-209+Cur group was characterized by a predominance of *Firmicutes*, *Bacillus*, and *Lactobacillus* bacterial groups, while the control group had the highest genus-level abundances of *Subdoligranulim*, *Oscillospira*, *Coprococcus*, *Barnesiella*, and *Ruminococcus*, as shown in Figure 2G. Harmful bacteria, including *Shigella* and *Bilophila*, were more prevalent in the BDE-209 group, but the beneficial genera *Oscillospira*, *Coprococcus*, *Barnesiella*, *Ruminococcus*, and *Lactobacillus* were less prevalent. When compared to the BDE-209 group, the abundance of these bacteria was somewhat improved by curcumin in the BDE-209+Cur group, approximating the control group.

### Effect of curcumin on liver injury in BDE-209-induced broilers

Light microscopic observation of H&E-stained liver tissues from the control and Cur groups (Figures 3A-a, 3A-c) revealed no abnormal pathological alterations. However, severe damage was observed in the liver tissues of the BDE-209 group, with disruption and even disappearance of the hepatic lobular structure and hepatocyte cords (black arrows), as well as hepatocyte edema, inflammatory cell infiltration (white arrow), and hemorrhage (Figure 3A-b, yellow arrow). The pathological damage score of this group was 10.33, which differed significantly from that of the control group (p<0.01). Curcumin treatment ameliorated the BDE-209-induced liver injury, as indicated by the restoration of normal hepatic lobular structure, a small amount of hepatocyte edema, and no significant inflammatory cell infiltration resulting in a striking decrease in the liver pathological score (p<0.01) (Figure 3A-d). Electron microscopy results (Figure 3A-e) showed the control group broiler chicken liver cells had a uniform distribution of nuclear and cytoplasmic materials, smooth nuclear membranes (red arrow), and rod-shaped mitochondria with clear cristae. In the BDE-209 group, liver cells had reduced chromatin in the nucleus, increased heterochromatin, nuclear shrinkage, thickened and highly irregular nuclear membranes (red arrow), swollen and vacuolated mitochondria (blue arrow), and disrupted mitochondrial cristae (Figure 3A-f). The proportion of disrupted mitochondrial cristae in the mitochondria increased significantly (p<0.01), and the average mitochondrial area was significantly smaller (p<0.05). In the Cur group, liver cells exhibited clear nuclear membranes with smooth edges, and the mitochondria were rod-shaped or spherical with well-arranged cristae, without apparent abnormalities (Figure 3A-g). In the BDE-209+Cur group, the liver cells had a relatively uniform distribution of nuclear and cytoplasmic materials and clear nuclear membranes with slightly wrinkled edges (red arrow), but the mitochondria had irregular shapes and a tendency to divide and fuse (Figure 3A-h). The number of mitochondria with crista disorder decreased significantly (p<0.01) and the average mitochondrial area increased (p<0.05) (Figures 3B, 3C).

Figures 3D–3H demonstrates that the BDE-209 group’s ALT, AST, LDH, AKP and GGT levels were significantly higher (p<0.01) than those of the control group. The BDE-209+Cur group’s ALT, AST, LDH, AKP and GGT levels were considerably lower (p<0.01) than those of the BDE-209 group. As seen in Figures 3I–3L, the BDE-209 group’s MDA content was significantly higher than that of the control group (p<0.01), whereas the concentrations of T-AOC, GSH, and CAT were significantly lower (p<0.01). The BDE-209+Cur group’s MDA content was significantly lower than that of the BDE-209 group (p<0.01), whereas the levels of T-AOC, GSH, and CAT were significantly higher (p<0.01).

### Effect of curcumin on the activation of hepatic inflammatory pathways induced by BDE-209

A notable increase in TLR4 protein expression was observed in the livers of broilers after being exposed to BDE-209 (Figures 4A, 4B, p<0.05). Furthermore, *MyD*88’s mRNA levels were markedly increased after exposure to BDE-209 (Figure 4C, p<0.01). The results demonstrated that exposure to BDE-209 triggered the NF-κB pathway, as indicated by a highly significant rise in the TNF-α protein expression level (p<0.01) and a considerable increase in the P-NF-κB/NF-κB ratio (p<0.05) (Figures 4A, 4D, 4E). Exposure to BDE-209 also led to a considerable upregulation of inflammatory factor expression in the liver (Figures 4F, 4G), with the *IL*-1β mRNA level increasing significantly (p<0.01), and the *IL*-6 mRNA content increasing highly significantly (p<0.05). However, the levels of *IL*-4 and *IL*-10 decreased significantly (Figures 4H, 4I, p<0.01). Curcumin significantly reversed the expression levels of these proteins and inflammatory factors (p<0.05, p<0.01).

The BDE-209 group showed higher P-P38/P38, P-ERK/ERK, and P-JNK/JNK ratios (p<0.01) than the control group, suggesting that the MAPK pathway was activated (Figures 4J–4M). Curcumin supplementation, however, reversed BDE-209’s induction of the MAPK pathway. As shown in Figure 4N, Oscillospira was negatively correlated with TLR4 (p<0.05) and positively correlated with IL-4 and IL-10 (p<0.01, p<0.05); Ruminococcus was negatively correlated with TLR4 and positively correlated with IL-4 and IL-10 (p<0.05); Bilophila was positively correlated with IL-1β and P-JNK/JNK (p<0.05), yet negatively correlated with IL-4 and IL-10 (p< 0.05).

### Effect of curcumin on endoplasmic reticulum stress in BDE-209-induced broiler livers

The immunohistochemistry results confirmed that the control and curcumin groups’ positive staining of the CHOP, GRP78, and TRAF2 proteins in the cytoplasm of the liver cells were less noticeable (Figures 5A–5F). The liver cells of the BDE-209 group had more cytoplasmic staining compared to the control group, as indicated by a statistically significant rise in the mean optical density value (p<0.01 or p<0.05). Conversely, both the average optical density value and the positive staining intensity decreased in the BDE-209+Cur group (p<0.01 or p<0.05). The BDE-209 group’s liver tissue had significantly higher levels of P-IRE1/IRE1, caspase-12 and the XBP1 protein (p<0.05) (Figures 5G–5J). On the other hand, the BDE-209+Cur group showed a significant drop in these values (p<0.01 or p<0.05). Furthermore, based on the RT-qPCR data (Figures 5K–5N). The mRNA expression levels of *GRP*78, *XBP*1, CHOP, and *TRAF*2 in the liver were significantly higher in the BDE-209 group compared to the control group (p<0.01). The mRNA expression levels of these genes decreased in the BDE-209+Cur group (p<0.01). Moreover, we analyzed the correlation between gut microbiota and ERS and found that *Lactobacillus* and *Oscillospira* were negatively correlated, whereas *Subdoligranulum* and *Bilophila* were positively correlated with ERS (Figure 5O).

## DISCUSSION

Curcumin is a natural antioxidant with metabolic regulatory, antibacterial, and anti-inflammatory properties [17]. On the contrary, BDE-209 is recognized for its toxicity and bioaccumulative potential, with the liver as its primary target for BDE-209-induced toxicity [18], suggesting a potential link between BDE-209 exposure and hepatic dysfunction. In this study, BDE-209 was found to induce intestinal damage and gut microbiota dysbiosis, which subsequently triggered oxidative stress, ERS, and inflammatory responses, ultimately leading to liver injury. However, curcumin alleviated BDE-209-induced hepatic damage in broilers by restoring intestinal barrier function, modulating the gut microbiota, and suppressing and alleviating stress responses and inflammatory reactions.

The intestine serves as a crucial organ for digestion and absorption, and intestinal morphological indices are commonly used to assess intestinal health status [19]. The intestinal epithelium, with TJs regulating polarity and paracellular permeability, forms the primary intestinal defensive barrier. The introduction of BDE-209 altered intestinal structure and induced an inflammatory response, potentially affecting the digestion and absorption in hens, thereby disrupting their growth and development and leading to weight loss [20]. BDE-209 downregulates TJ-related mRNA and protein expression, damaging intestinal structure (e.g., villus collapse/shedding) and impairing barrier function, potentially exacerbating its adverse effects. BDE-209 not only increased the feed-to-gain ratio but also elevated the liver weight and liver index, thereby negatively affecting the growth and development of broiler chickens. Xun et al. [21] reported that supplementation with curcumin decreased the feed-to-gain ratio, improved intestinal structure, and lowered inflammatory cytokine levels. In addition, studies have shown that curcumin can significantly increase the levels of ZO-1 and occludin in rats with type 2 diabetes [22]. Therefore, curcumin can protect the structural integrity of the broiler intestine, alleviate intestinal inflammation, and mitigate the adverse effects of BDE-209 on the growth and development of broiler chickens.

The gut microbiota acts as a cornerstone for maintaining the host’s physiological homeostasis, profoundly influencing both health and disease by regulating metabolism, immunity, and barrier function [23]. As the gut microbiota is extremely sensitive to environmental pollutants, it is a vulnerable target affected by antibiotics, heavy metals, and POPs [24]. Since BDE-209 is a type of POP, and acute exposure to BDE-209 can lead to gut microbiota dysbiosis in zebrafish [25], it is not surprising that BDE-209 significantly altered the β-diversity of the gut microbiota, decreasing the abundance of Firmicutes and beneficial bacteria (including *Lactobacillus*, *Oscillospira*, and *Ruminococcus*) while increasing the abundance of *Proteobacteria*, *Shigella*, and *Bilophila* in broilers. However, curcumin treatment reversed these effects. *Shigella*, a *Proteobacteria*, is linked to chronic diseases, causes inflammation, and is a gut dysbiosis marker [26]. In contrast, *Lactobacillus* maintains gut health by inhibiting inflammatory pathways and improving the intestinal barrier [27]. *Lactobacillus* also has hepatoprotective effects [28]. Ruminococcaceae-produced SCFAs like acetate support health, and butyrate alleviates hepatic steatosis by restoring gut balance and reduces liver inflammation via the NF-κB pathway [29]. Correlation analysis revealed that *Oscillospira* and *Ruminococcus* were negatively correlated with TLR4 but positively correlated with IL-4 and IL-10. In contrast, *Bilophila* showed positive correlations with IL-1β and P-JNK/JNK, and negative correlations with IL-4 and IL-10. These results suggest that BDE-209 exposure disrupts the intestinal microbial balance by decreasing beneficial bacteria and increasing harmful bacteria, thereby promoting hepatic and intestinal inflammation and impairing intestinal barrier function.

When the gut microbiota is dysregulated, the intestinal barrier function is impaired, and bacteria and their metabolites in the intestine, such as lipopolysaccharide (LPS), leak into the bloodstream and reach the liver [30]. This subsequently activates immune cells and triggers pathways such as NADPH oxidase, generating a large amount of ROS, thereby inducing systemic and hepatic oxidative stress, as confirmed in our study. Curcumin contains various functional antioxidant groups, including the β-diketone moiety, carbon-carbon double bonds, and phenyl rings. It can scavenge lipid radicals in cell membranes and convert into phenoxyl radicals, thus being regarded as a potent fat-soluble antioxidant [31]. This aligns with our findings, indicating that the supplementation of curcumin can alleviate BDE-209-induced hepatic oxidative stress.

Moreover, ROS can directly attack proteins within the endoplasmic reticulum, leading to their misfolding. It can also disrupt calcium ion homeostasis in the endoplasmic reticulum and interfere with chaperone functions, thereby inducing or exacerbating ERS [32]. The ER, which is well-developed in hepatocytes, is implicated in liver-related diseases, where many external stimuli, including radiation, hypoxia, toxic chemicals, and microbial infections, can impair ER function and induce ERS [33]. Studies have found that exposure to BDE-209 significantly elevates the expression levels of ERS-related factors such as P-IRE1/IRE1 and CHOP, triggering ERS in the liver [34]. Additionally, caspase-12 and XBP1 activation by BDE-209 worsens liver ERS. In livers, curcumin can suppress inflammation, apoptosis, and ERS [35] and our results support these findings with curcumin effectively inhibiting hepatic ERS by reducing the expression of ERS-related factors such as P-IRE1/IRE1 and CHOP.

Oxidative stress and ERS synergistically disrupt cellular homeostasis. When the accumulated damage exceeds the cell’s repair capacity, danger signals are triggered, activating core inflammatory pathways such as NF-κB and thereby inducing systemic inflammation [36]. Within the TLR4/MyD88/NF-κB/MAPK pathway, which is crucial for inflammation regulation [37], the NF-κB protein is recognized as an important regulator of the ERS signaling pathway. When TLR4 triggers inflammatory pathways through molecules such as MyD88, pro-inflammatory proteins are released [38], inducing inflammation.

Hepatic inflammatory responses directly attack hepatocytes by releasing pro-inflammatory factors and recruiting infiltrating immune cells, leading to structural pathological damage such as hepatocyte necrosis, apoptosis, and impaired bile excretion. This damage results in hepatocyte membrane rupture, leakage of intracellular enzymes, and dysfunction of bile ducts, directly manifested as elevated key liver function indicators in the blood, such as ALT, AST, GGT, and bilirubin [39]. This aligns with the hepatic pathological damage and abnormal elevation of liver function indicators induced by BDE-209 observed in our study. In addition to its potent antioxidant properties, curcumin also exhibits significant anti-inflammatory effects, playing a key role in mitigating liver inflammation and reducing the risk of tissue damage. Curcumin regulates the NF-κB pathway, inhibits the release of pro-inflammatory cytokines and chemokines, and modulates the inflammatory microenvironment in the liver, thereby preventing tissue damage [40]. This has also been confirmed in our study, indicating that curcumin can alleviate BDE-209-induced liver damage in broilers. It should be noted that while a strong association was observed between gut microbiota restoration and liver protection, the present study does not prove direct causality. Further experiments (e.g., FMT or antibiotic depletion) are needed to determine whether gut microbiota changes are a causal driver of curcumin’s hepatoprotective effects. Curcumin has low oral bioavailability in poultry due to poor solubility and rapid metabolism, which often necessitates higher dietary doses. The 0.3 g/kg dose used in this study, while effective, is relatively high for commercial application. However, crude turmeric powder (containing 2%–5% curcumin) may offer a more cost-effective alternative, and bioavailability-enhancing strategies (e.g., piperine co-administration) could achieve similar efficacy at lower doses.

## CONCLUSION

In short, we have demonstrated for the first time the protective effect of curcumin against BDE-209-induced liver injury. Curcumin can serve as a potential therapeutic agent for preventing and treating liver injury caused by environmental pollutants such as BDE-209, and its protective effects are associated with repairing intestinal barrier function, restoring the gut microbiota structure, alleviating oxidative stress and ERS, as well as modulating the hepatic TLR4/MyD88/NF-κB/MAPK pathway to suppress inflammatory responses.

## Figures and Tables

**Figure 1:**
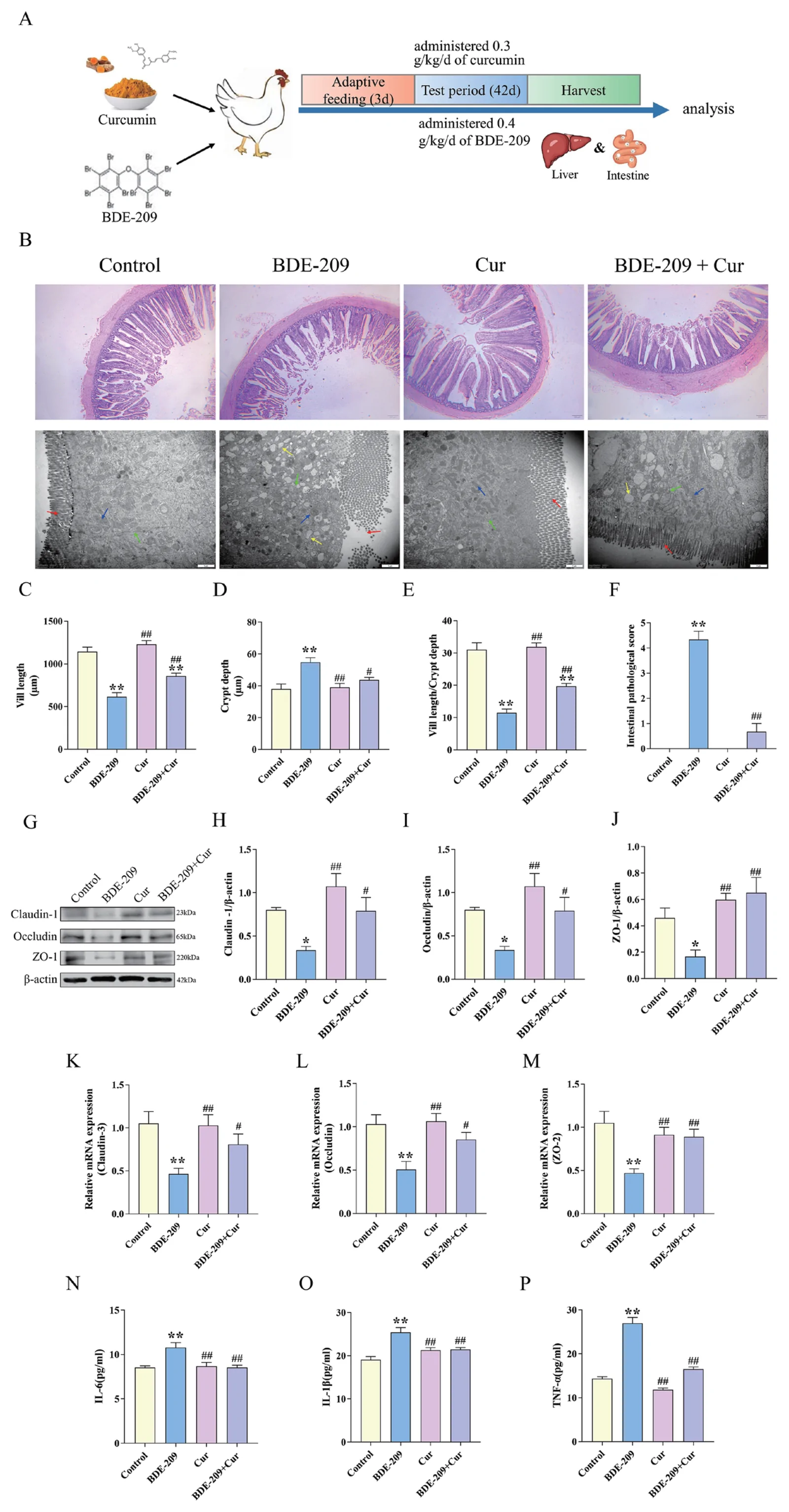
Effects of curcumin on intestinal histopathology, intestinal barrier function, and intestinal inflammation of broilers. (A) Experimental period chart. (B) Hematoxylin-eosin staining of the intestinal section (100×, scale bar = 200 μm) and transmission electron microscopy (15,000×, scale bar = 1 μm) to detect intestinal tissue. Red arrow: intestinal villus; green arrow: tight junction (TJ) structure; blue arrow: mitochondria; yellow arrow: vacuoles. (C) Intestinal villus height. (D) Intestinal crypt depth. (E) Intestinal villus height/Intestinal crypt depth. (F) Intestinal pathological score. (G) Western blot of the expression of Claudin-1, Occludin, and ZO-1. (H–J) Relative expressions of Claudin-1, Occludin, and ZO-1. (K–M) mRNA expression levels of Claudin-3, Occludin, and ZO-2. (N–P) Levels of IL-6, IL-1β, and TNF-α. Data are presented as mean±SD. Compared with the control group, * p<0.05, ** p<0.01; compared with the BDE-209 group, ^#^ p<0.05, ^##^ p<0.01. SD, standard deviation.

**Figure 2:**
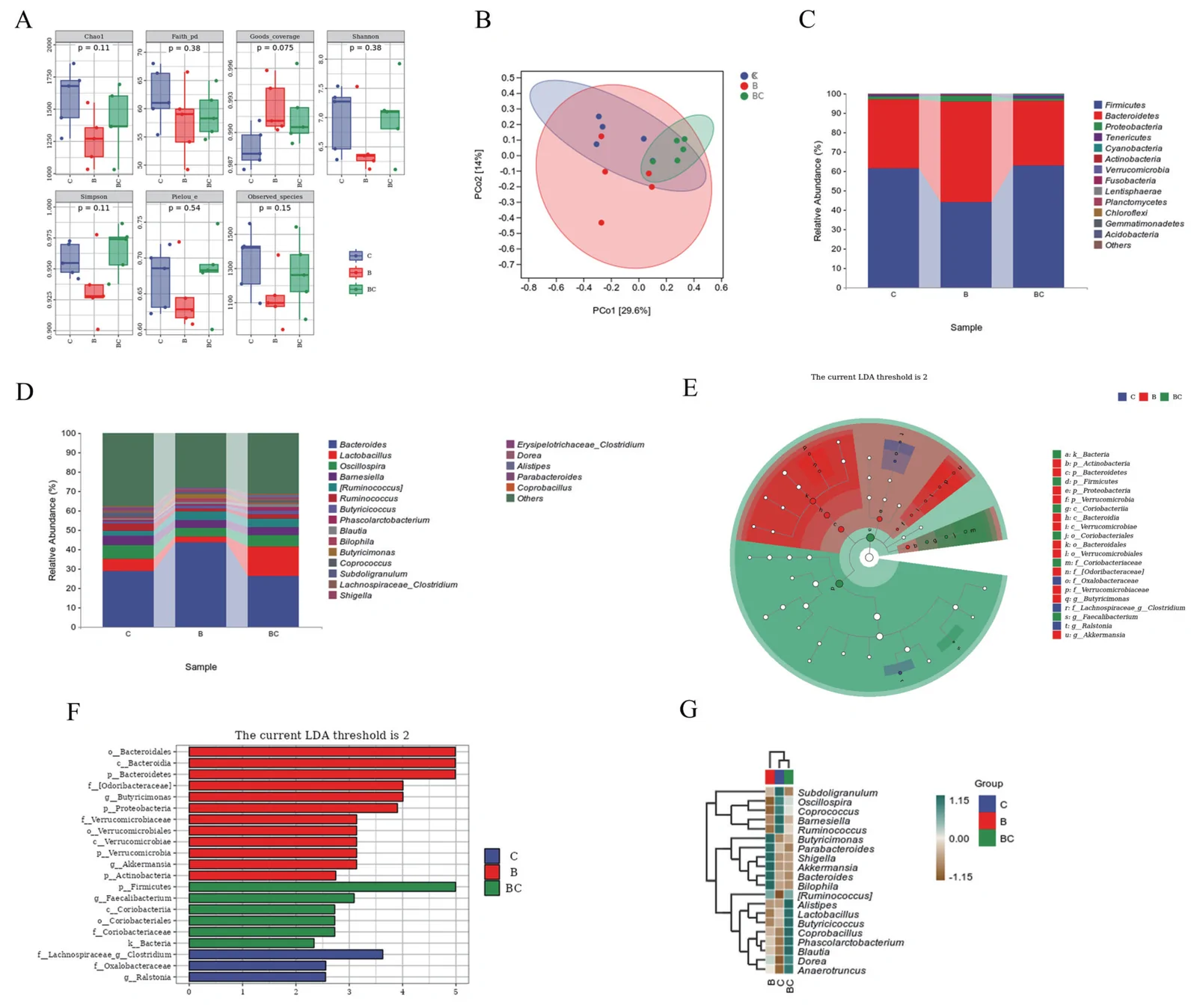
Curcumin alleviates BDE-209-induced intestinal microflora disorders in broilers. Group C is the control group, group B is the BDE-209 group, and group BC is the BDE-209+Cur group. (A) The alpha diversity among groups. (B) The beta diversity among groups. (C) The composition of gut microbiota of community bar plot analysis at the phylum level. (D) The composition of gut microbiota of community bar plot analysis at the genus level. (E) Lefse taxonomic cladogram. Colored nodes from the outer ring to the inner ring represent the relationship between the intestinal flora at the genus level and the phylum level. (F) Groups with LDA scores >2. (G) The clustering diagram shows the differential microbial communities among the top 20 ranked genera. Gut microbiota was detected in all cases.

**Figure 3:**
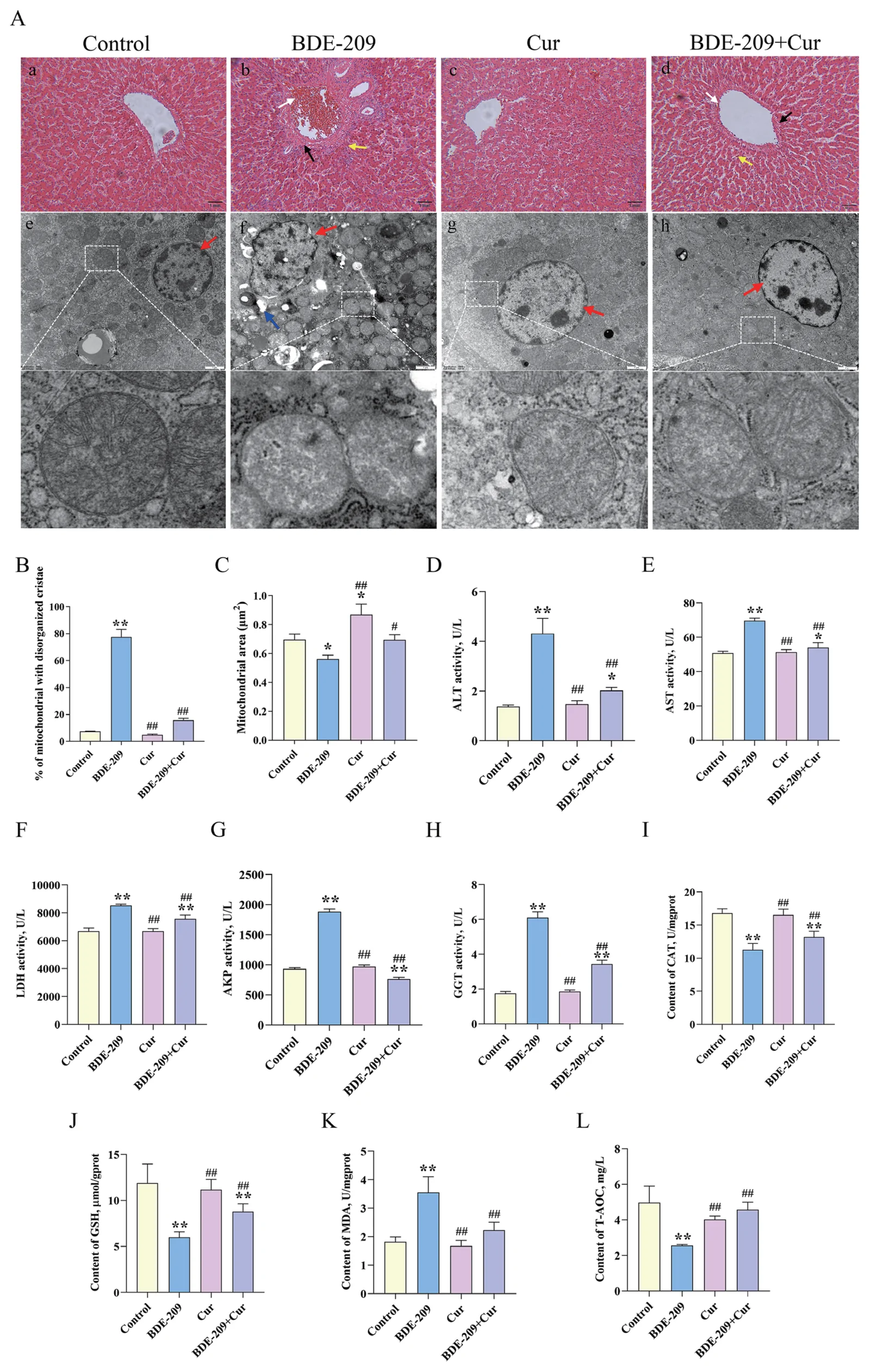
Effects of curcumin on liver histopathology, liver function, and oxidative stress of broilers. (A) Hematoxylin-eosin staining of liver tissue (200×, scale bar = 50 μm) and transmission electron microscopy (15,000×, scale bar = 1 μm) to detect liver tissue. White arrow: inflammatory infiltration; yellow arrow: hemorrhage; black arrow: the hepatocytes were ruptured or missing; red arrow: cell nucleus; green arrow: mitochondria; blue arrow: cell vacuolization. (B) The proportion of mitochondria with disorganized cristae. (C) Mitochondrial area (μm^2^). (D–H) Liver function index: (D) ALT, (E) AST, (F) LDH, (G) AKP, and (H) GGT. (I–L) Indicators of liver oxidative stress, (I) CAT, (J) GSH, (K) MDA, and (L) T-AOC. Data are presented as mean±SD. Compared with the control group, * p<0.05, ** p<0.01; compared with the BDE-209 group, ^#^ p<0.05, ^##^ p<0.01. ALT, alanine aminotransferase; AST, aspartate aminotransferase; LDH, lactate dehydrogenase; GGT, gamma-glutamyl transferase; CAT, catalase; GSH, glutathione; MDA, malondialdehyde; T-AOC, total antioxidant capacity; SD, standard deviation.

**Figure 4:**
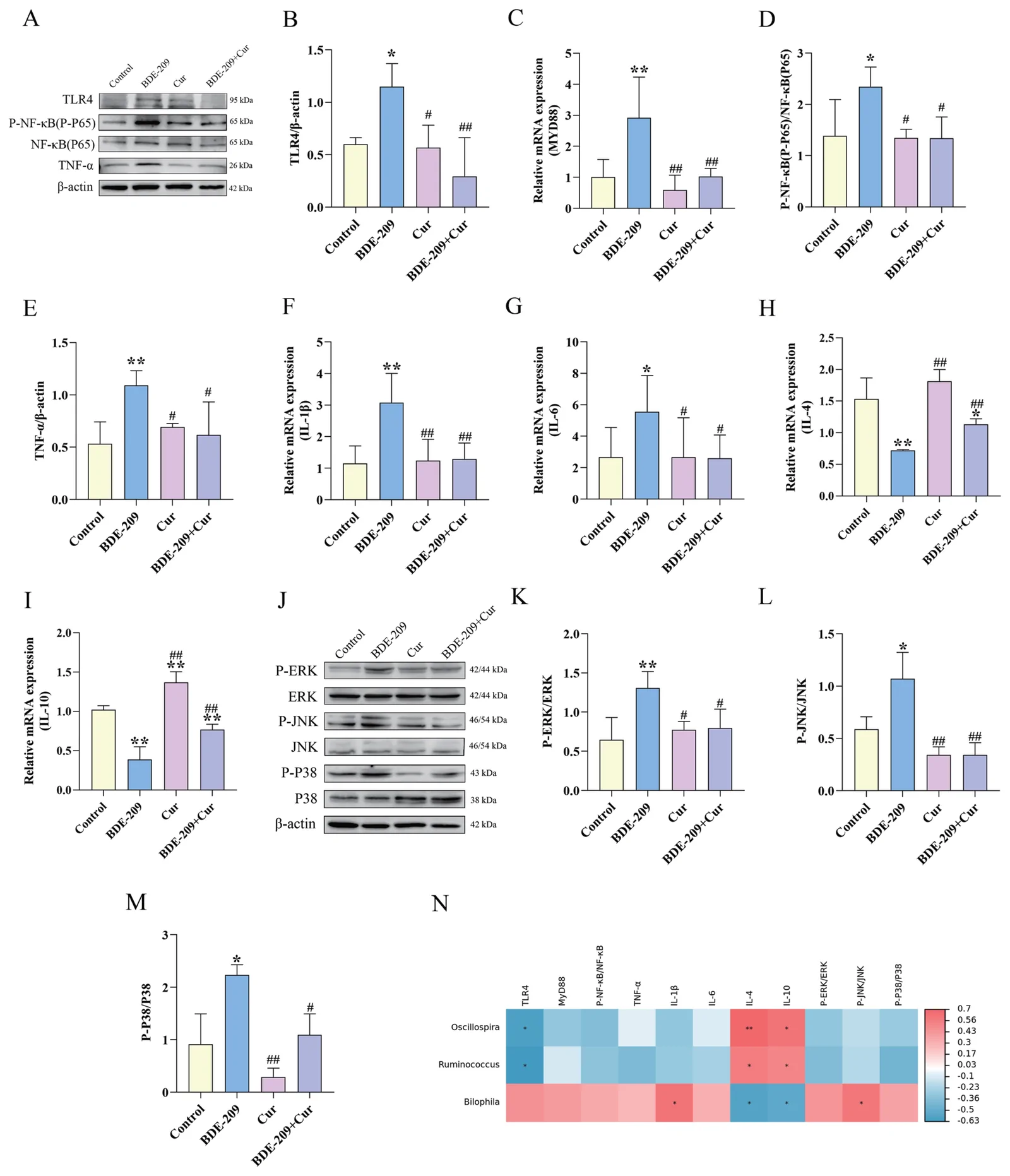
Curcumin inhibits the activation of hepatic inflammatory pathways induced by BDE-209 in the liver tissue of broilers. (A, B) The protein expression of TLR4 in liver tissue. (C) Expression of MyD88 mRNA in liver tissue. (D) The ratio of P-NF-κB to NF-κB in the liver. (E) The protein expression of TNF-α in liver tissue. (F–I) Expressions of (F) IL-1β, (G) IL-6, (H) IL-4, (I) IL-10 mRNA in liver tissue. (J–M) Expression of MAPK pathway-related proteins in the liver. (N) Heatmap of the relationship between altered gut microbes and inflammatory pathway factors in broilers treated with BDE-209. The color key designates the strength of correlation (r-value). Dark red indicates a more positive correlation, dark blue indicates a more negative correlation, and white indicates no correlation. Correlations with p<0.05 and r>0.5 are regarded as related and significant. Data are presented as mean±SD. Compared with the control group, * p<0.05, ** p<0.01; compared with the BDE-209 group, ^#^ p<0.05, ^##^ p<0.01. SD, standard deviation.

**Figure 5:**
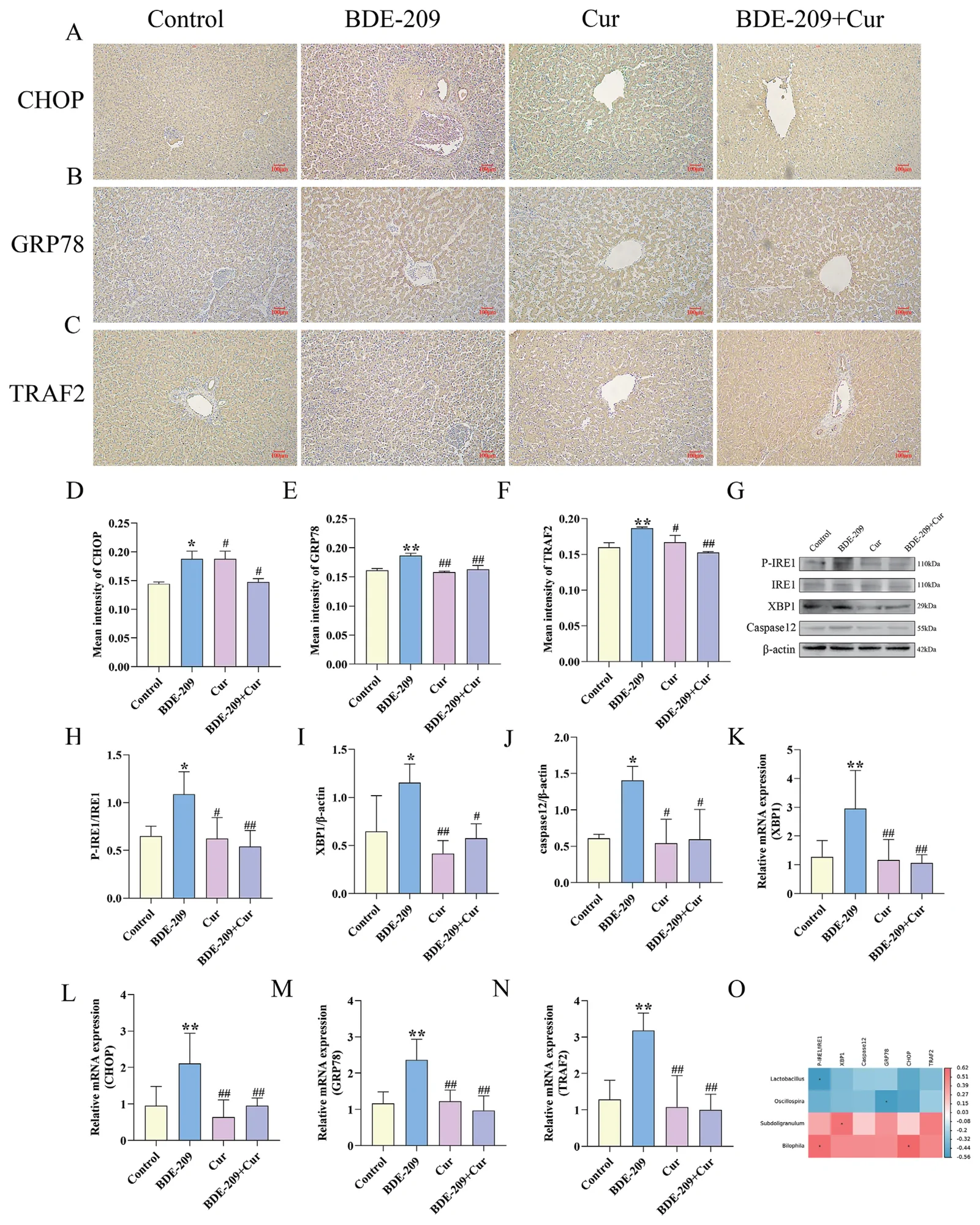
Effects of curcumin on the endoplasmic reticulum stress (ERS) pathway and liver immunohistochemistry of broilers. (A, D) CHOP expression in liver tissue. (B, E) GRP78 expression in liver tissue. (C, F) TRAF2 expression in liver tissue. (G) Western blot of protein expression related to the ERS pathway in the liver. (H) The ratio of P-IRE1 to IRE1. (I) Protein expression of XBP1. (J) Protein expression of Caspase-12. (K–N) The mRNA content of genes related to the ERS pathway in the liver: (K) XBP1, (L) CHOP, (M) GRP78, (N) TRAF2. (O) Heatmap of the relationship between altered gut microbes and ERS pathway factors in broilers treated with BDE-209. The color key indicates the strength of correlation (r-value). Dark red specifies a more positive correlation, dark blue indicates a more negative correlation, and white indicates no correlation. Correlations with p<0.05 and r>0.5 are regarded as related and significant. Data are presented as mean±SD. Compared with the control group, * p<0.05, ** p<0.01; compared with the BDE-209 group, ^#^ p<0.05, ^##^ p<0.01. SD, standard deviation.

**Table 1:** RT-qPCR primer sequences

Primer name	Primer sequences (5′→ 3′)	Amplicon size (bp)	Annealing temperature	GenBank accession numbers
GAPDH	F: AGTGAAGGCTGCTGCTGATGG	105	63	NM_204305.2
	R: TCAAAGGTGGAGGAATGGCTGTC			
Occludin	F: CTGCTCTGCCTCATCTGCTTCTTC	143	63	NM_205128.1
	R: CCATCCGCCACGTTCTTCACC			
*Claudin-3*	F: CTTCATCGGCAACAACATCGTGAC	113	63	NM_204202.2
	R: CCAGCATGGAGTCGTACACCTTG			
*ZO-2*	F: GCAGTGATGGAGAGGAGGAGGAG	83	63	XM_046934789.1
	R: TCTGGGTGGCTGTCCGTAGTAAC			
*IL-1β*	F: CAGAAGAAGCCTCGCCTGGATTC	133	64	NM_204524.2
	R: GCCTCCGCAGCAGTTTGGTC			
*IL-6*	F: GAGGTTGGGCTGGAGGAGGAG	130	63	NM_204628.2
	R: TCTCGCACACGGTGAACTTCTTG			
*IL-4*	F: GTGCCCACGCTGTGCTTAC	82	61	NM_001007079.2
	R: AGGAAACCTCTCCCTGGATGTC			
*IL-10*	F: CGCTGTCACCGCTTCTTCA	88	60	NM_001004414.4
	R: TCCCGTTCTCATCCATCTTCTC			
*GRP78*	F: TCCTGCTCCTCGTGGTGTCC	148	63	NM_205491.2
	R: CTCCTCTGGTGTTAGCCGATTCTG			
*TRAF2*	F: AAGGTTGTCGGCTGTGCTGAG	133	63	XM_015279621.3
	R: GCTTCAGGTCTCCAGATCCACTC			
CHOP	F: AGCTGGATGAGACACTGAATGCAG	149	63	XM_040693765.2
	R: ACGCTTCCGCTTTGTCCTCTG			
*MyD88*	F: AAGGTGTCGGAGGATGGTGGTC	120	63	XM_046921393.1
	R: GGAATCAGCCGCTTGAGACGAG			
*XBP1*	F: GGAGTGCGAGTCTACGGATGTG	145	62	NM_001006192.2
	R: TGTGGAGGTTGTCAGGAATGGTG			

RT-qPCR, reverse transcription-quantitative polymerase chain reaction.

**Table 2:** Dilution concentration of antibody for WB analysis

Antibodies	Dilution ratio	Vendor	Catalog number	Host species
ZO-1	1:1,000	Wanleibio (Shenyang, China)	WL03419	Rabbit
Claudin-1	1:1,000	Wanleibio (Shenyang, China)	WL03073	Rabbit
Occludin	1:1,000	Wanleibio (Shenyang, China)	WL01996	Rabbit
TLR4	1:1,000	Wanleibio (Shenyang, China)	WL00196	Rabbit
NF-κB P65	1:1,000	Wanleibio (Shenyang, China)	WL01980	Rabbit
P-NF-κB P65	1:500	Wanleibio (Shenyang, China)	WL02169	Rabbit
TNF-α	1:1,000	Wanleibio (Shenyang, China)	WL01581	Rabbit
ERK	1:500	Wanleibio (Shenyang, China)	WL01770	Rabbit
p-ERK	1:500	Wanleibio (Shenyang, China)	WLP1512	Rabbit
p38	1:1,000	Wanleibio (Shenyang, China)	WL00764	Rabbit
p-p38	1:500	Wanleibio (Shenyang, China)	WLP1576	Rabbit
JNK	1:1,000	Diagbio (Hangzhou, China)	DGR11169	Rabbit
p-JNK	1:500	Wanleibio (Shenyang, China)	WL01813	Rabbit
XBP1	1:1,000	Diagbio (Hangzhou, China)	DGR16363	Rabbit
IRE1	1:1,000	Wanleibio (Shenyang, China)	WL02562	Rabbit
p-IRE1	1:1,000	Wanleibio (Shenyang, China)	WL05299	Rabbit
Caspase-12	1:1,000	Wanleibio (Shenyang, China)	WL03268	Rabbit
β-actin	1:500	Wanleibio (Shenyang, China)	WL01372	Rabbit

**Table 3:** Growth performance indexes of broilers

	Control	BDE-209	Cur	BDE-209+Cur
Initial weight (g)	49.64±1.61	50.41±2.07	50.84±2.34	51.04±1.72
Daily intake(g)	85.01±0.75	83.58±0.93^*^	85.28±0.48	84.27±0.02
Average daily gain (g)	51.38±3.65	44.16±2.36^**^	53.22±2.70^##^	50.40±2.19^##^
Final weight (g)	2,053.5±142.32	1,772.6±92.67^**^	2,126.8±104.77^##^	2,016.7±84.82^##^
Liver weight (g)	37.9±1.30	44.0±4.20^**^	37.0±1.60^##^	40.3±3.70^*#^
Liver index (%)	1.80±0.15	2.38±0.37^**^	1.78±0.12^##^	2.00±0.15^*##^

Data are presented as mean±SD.

Compared with the control group,

* p<0.05,

** p<0.01; compared with the BDE-209 group,

# p<0.05,

## p<0.01.

Cur, curcumin; SD, standard deviation.

## Data Availability

Upon reasonable request, the datasets of this study can be available from the corresponding author.

## References

[1] Burgos Melo HD de Souza-Araujo J Cardoso R PBDE concentrations in wastes from construction and demolition, soft furnishings, and end-of-life vehicles in Brazil Environ Res 2025 287 122963 10.1016/j.envres.2025.122963 41027492

[2] Xu J Ge F Yu J Levels, distribution and ecological risk assessment of PBDEs in soils and plants around the engineering plastics factory Bull Environ Contam Toxicol 2024 112 75 10.1007/s00128-024-03891-0 38733395

[3] Liu Z Tang X Lv M A toxicokinetic study of decabromodiphenyl ether (BDE-209) in marine planktonic food web-adsorption/absorption kinetics, biotransformation, transfer patterns along the food chain J Hazard Mater 2025 496 139467 10.1016/j.jhazmat.2025.139467 40784112

[4] Li B Wang J Hu G Bioaccumulation behavior and human health risk of polybrominated diphenyl ethers in a freshwater food web of typical shallow lake, Yangtze River Delta Int J Environ Res Public Health 2023 20 2671 10.3390/ijerph20032671 PMC9916311 36768037

[5] Fernandes AR Lake IR Dowding A The transfer of environmental contaminants (brominated and chlorinated dioxins and biphenyls, PBDEs, HBCDDs, PCNs and PFAS) from recycled materials used for bedding to the eggs and tissues of chickens Sci Total Environ 2023 892 164441 10.1016/j.scitotenv.2023.164441 37245822

[6] Yang Y Zhu X Rao Q Liu Z Yang J Zhao Z Toxicokinetics and edible tissues-specific bioaccumulation of decabrominated diphenyl ethers (BDE-209) after exposure to the broilers Ecotoxicol Environ Saf 2022 248 114324 10.1016/j.ecoenv.2022.114324 36434998

[7] Yang Y Jin Y Zhu X Rao Q Zhao Z Yang J Hepatotoxicity evaluation and possible mechanisms of decabrominated diphenyl ethers (BDE-209) in broilers: oxidative stress, inflammatory, and transcriptomics Ecotoxicol Environ Saf 2023 264 115460 10.1016/j.ecoenv.2023.115460 37696076

[8] Li D Li Z Dong L Coffee prevents IQ-induced liver damage by regulating oxidative stress, inflammation, endoplasmic reticulum stress, autophagy, apoptosis, and the MAPK/NF-κB signaling pathway in zebrafish Food Res Int 2023 169 112946 10.1016/j.foodres.2023.112946 37254370

[9] Zhao L Liang J Chen F High carbohydrate diet induced endoplasmic reticulum stress and oxidative stress, promoted inflammation and apoptosis, impaired intestinal barrier of juvenile largemouth bass (Micropterus salmoides) Fish Shellfish Immunol 2021 119 308 17 10.1016/j.fsi.2021.10.019 34662728

[10] Zhao Z Zong Y Zhang W Wang K Yang Y Li F Nuciferine alleviates non-alcoholic steatohepatitis by restoring endoplasmic reticulum stress Exp Cell Res 2025 449 114599 10.1016/j.yexcr.2025.114599 40339780

[11] Dehzad MJ Ghalandari H Nouri M Askarpour M Antioxidant and anti-inflammatory effects of curcumin/turmeric supplementation in adults: a GRADE-assessed systematic review and dose–response meta-analysis of randomized controlled trials Cytokine 2023 164 156144 10.1016/j.cyto.2023.156144 36804260

[12] Chiu YH Chou WL Ko MC Liao JC Huang TH Curcumin mitigates obesity-driven dysbiosis and liver steatosis while promoting browning and thermogenesis in white adipose tissue of high-fat diet-fed mice J Nutr Biochem 2025 143 109920 10.1016/j.jnutbio.2025.109920 40239823

[13] Hu H Lin A Kong M Intestinal microbiome and NAFLD: molecular insights and therapeutic perspectives J Gastroenterol 2020 55 142 58 10.1007/s00535-019-01649-8 PMC6981320 31845054

[14] Cheng P Ishfaq M Yu H Curcumin ameliorates duodenal toxicity of AFB1 in chicken through inducing P-glycoprotein and downregulating cytochrome P450 enzymes Poult Sci 2020 99 7035 45 10.1016/j.psj.2020.09.055 PMC7705060 33248620

[15] Sneed RA Grimes SD Schultze AE Brown AP Ganey PE Bacterial endotoxin enhances the hepatotoxicity of allyl alcohol Toxicol Appl Pharmacol 1997 144 77 87 10.1006/taap.1997.8115 9169072

[16] Shen L Zhou Y Wu X TREM1 blockade ameliorates lipopolysaccharide-induced acute intestinal dysfunction through inhibiting intestinal apoptosis and inflammation response BioMed Res Int 2021 2021 6635452 10.1155/2021/6635452 PMC8068534 33954188

[17] Kępińska-Pacelik J Biel W Turmeric and curcumin: health-promoting properties in humans versus dogs Int J Mol Sci 2023 24 14561 10.3390/ijms241914561 PMC10572432 37834009

[18] Chen S Che S Li S Ruan Z The combined impact of decabromodiphenyl ether and high fat exposure on non-alcoholic fatty liver disease in vivo and in vitro Toxicology 2021 464 153015 10.1016/j.tox.2021.153015 34757160

[19] Li W Liu B Liu Z Effect of dietary histamine on intestinal morphology, inflammatory status, and gut microbiota in yellow catfish (Pelteobagrus fulvidraco) Fish Shellfish Immunol 2021 117 95 103 10.1016/j.fsi.2021.07.017 34284110

[20] Liu Z Lei H Tang R Effects of decabrominated diphenyl ether exposure on growth, meat characteristics and blood profiles in broilers Animals 2021 11 565 10.3390/ani11020565 PMC7926798 33671620

[21] Xun W Shi L Zhou H Effects of curcumin on growth performance, jejunal mucosal membrane integrity, morphology and immune status in weaned piglets challenged with enterotoxigenic Escherichia coli Int Immunopharmacol 2015 27 46 52 10.1016/j.intimp.2015.04.038 25937483

[22] Huang J Guan B Lin L Wang Y Improvement of intestinal barrier function, gut microbiota, and metabolic endotoxemia in type 2 diabetes rats by curcumin Bioengineered 2021 12 11947 58 10.1080/21655979.2021.2009322 PMC8810160 34818970

[23] Scazzocchio B Minghetti L D’Archivio M Interaction between gut microbiota and curcumin: a new key of understanding for the health effects of curcumin Nutrients 2020 12 2499 10.3390/nu12092499 PMC7551052 32824993

[24] Chen X Zhang Z Hsueh Y Interactions between environmental pollutants and gut microbiota: a review connecting the conventional heavy metals and the emerging microplastics Environ Res 2025 269 120928 10.1016/j.envres.2025.120928 39855410

[25] Wang J Liu C Wang SP BDE-209-induced genotoxicity, intestinal damage and intestinal microbiota dysbiosis in zebrafish (Danio rerio) Sci Total Environ 2023 905 167009 10.1016/j.scitotenv.2023.167009 37704147

[26] Muttiah B Hanafiah A Gut microbiota and cardiovascular diseases: unraveling the role of dysbiosis and microbial mMetabolites Int J Mol Sci 2025 26 4264 10.3390/ijms26094264 PMC12072866 40362500

[27] Wu Y Jha R Li A Probiotics (Lactobacillus plantarum HNU082) supplementation relieves ulcerative colitis by affecting intestinal barrier functions, immunity-related gene expression, gut microbiota, and metabolic pathways in mice Microbiol Spectr 2022 10 e01651 22 10.1128/spectrum.01651-22 PMC9769980 36321893

[28] Saeedi BJ Liu KH Owens JA Gut-resident Lactobacilli activate hepatic Nrf2 and protect against oxidative liver injury Cell Metab 2020 31 956 68.E5 10.1016/j.cmet.2020.03.006 PMC7329068 32213347

[29] Yi G Sun W Zhang X Soybean genistin-driven gut microbiota-derived butyrate synthesis activates the SIRT1 signaling pathway to ameliorate metabolic fatty liver Free Radic Biol Med 2025 239 336 51 10.1016/j.freeradbiomed.2025.07.053 40752635

[30] Li C Cai C Wang C Chen X Zhang B Huang Z Gut microbiota-mediated gut-liver axis: a breakthrough point for understanding and treating liver cancer Clin Mol Hepatol 2025 31 350 81 10.3350/cmh.2024.0857 PMC12016628 39659059

[31] Farzaei MH Zobeiri M Parvizi F Curcumin in liver diseases: a systematic review of the cellular mechanisms of oxidative stress and clinical perspective Nutrients 2018 10 855 10.3390/nu10070855 PMC6073929 29966389

[32] Tang Y Zhou X Cao T Endoplasmic reticulum stress and oxidative stress in inflammatory diseases DNA Cell Biol 2022 41 924 34 10.1089/dna.2022.0353 36356165

[33] Zhang J Guo J Yang N Huang Y Hu T Rao C Endoplasmic reticulum stress-mediated cell death in liver injury Cell Death Dis 2022 13 1051 10.1038/s41419-022-05444-x PMC9763476 36535923

[34] Chen S Che S Li S Wan J Ruan Z High-fat diet exacerbated decabromodiphenyl ether-induced hepatocyte apoptosis via intensifying the transfer of Ca2+ from endoplasmic reticulum to mitochondria Environ Pollut 2022 292 118297 10.1016/j.envpol.2021.118297 34624399

[35] Wu L Zhao P Wu P Curcumin attenuates ochratoxin A and hypoxia co-induced liver injury in grass carp (Ctenopharyngodon idella) by dual targeting endoplasmic reticulum stress and apoptosis via reducing ROS content J Anim Sci Biotechnol 2024 15 131 10.1186/s40104-024-01089-2 PMC11451059 39363374

[36] Zha Z Xiao D Liu Z Endoplasmic reticulum stress induces ROS production and activates NLRP3 inflammasome via the PERK-CHOP signaling pathway in dry eye disease Invest Ophthalmol Vis Sci 2024 65 34 10.1167/iovs.65.14.34 PMC11668352 39699913

[37] Sun Z He W Meng H Li P Qu J Endoplasmic reticulum stress in acute lung injury and pulmonary fibrosis FASEB J 2024 38 e70232 10.1096/fj.202401849RR 39651914

[38] Schlichter Kadosh Y Muthuraman S Kushmaro A Kumar RS Gopas J NF-κB inhibitory activity of the di-hydroxy derivative of piperlongumine (PL-18) J Immunol Res 2025 2025 9915695 10.1155/jimr/9915695 PMC11735059 39822305

[39] Taru V Szabo G Mehal W Reiberger T Inflammasomes in chronic liver disease: hepatic injury, fibrosis progression and systemic inflammation J Hepatol 2024 81 895 910 10.1016/j.jhep.2024.06.016 PMC11881887 38908436

[40] Ruiz de Porras V Figols M Font A Pardina E Curcumin as a hepatoprotective agent against chemotherapy-induced liver injury Life Sci 2023 332 122119 10.1016/j.lfs.2023.122119 37741319

